# The Impact of Bioactive Surfaces in the Early Stages of Osseointegration: An *In Vitro* Comparative Study Evaluating the HAnano® and SLActive® Super Hydrophilic Surfaces

**DOI:** 10.1155/2020/3026893

**Published:** 2020-09-13

**Authors:** Rodrigo A. da Silva, Geórgia da Silva Feltran, Marcel Rodrigues Ferreira, Patrícia Fretes Wood, Fabio Bezerra, Willian F. Zambuzzi

**Affiliations:** ^1^Lab. of Bioassays and Cellular Dynamics, Department of Chemical and Biological Sciences, Institute of Biosciences, UNESP-São Paulo State University, 18618-970, Botucatu, São Paulo, Brazil; ^2^School of Dentistry, University of Taubaté, 12020-340, Taubaté, São Paulo, Brazil; ^3^Program in Environmental and Experimental Pathology, Paulista University, São Paulo, 04026-002 São Paulo, Brazil

## Abstract

There is an increased effort on developing novel and active surfaces in order to accelerate their osteointegration, such as nanosized crystalline hydroxyapatite coating (HAnano®). To better understand the biological behavior of osteoblasts grown on HAnano® surface, the set of data was compared with SLActive®, a hydrophilic sandblasted titanium surface. Methodologically, osteoblasts were seeded on both surfaces up to 72 hours, to allow evaluating cell adhesion, viability, and set of genes encoding proteins related with adhesion, proliferation, and differentiation. Our data shows HAnano® displays an interesting substrate to support cell adhesion with typical spread morphologic cells, while SLActive®-adhering cells presented fusiform morphology. Our data shows that the cellular adhesion mechanism was accompanied with upexpression of *integrin β1*, *Fak*, and *Src*, favoring the assembling of focal adhesion platforms and coupling cell cycle progression (upmodulating of *Cdk2*, *Cdk4*, and *Cdk6* genes) in response to HAnano®. Additionally, both bioactive surfaces promoted osteoblast differentiation stimulus, by activating *Runx2*, *Osterix*, and *Alp* genes. Although both surfaces promoted *Rankl* gene expression, Opg gene expression was higher in SLActive® and this difference reflected on the *Rankl*/*Opg* ratio. Finally, Caspase1 gene was significantly upmodulated in response to HAnano® and it suggests an involvement of the inflammasome complex. Collectively, this study provides enough evidences to support that the nanohydroxyapatite-coated surface provides the necessary microenvironment to drive osteoblast performance on dental implants and these stages of osteogenesis are expected during the early stages of osseointegration.

## 1. Introduction

In dentistry, biomaterials are related with a wide spectrum of applications such as prostheses and implants in reparative procedures. Considering the implantology field, commercial pure titanium and titanium alloys have been widely applied in edentulism treatment presenting good biological outcomes [1]. Although titanium assemblies have adequate biological and physicochemical properties, there is an increase effort on developing novel and active surfaces in order to accelerate their osteointegration [2]. Osteointegration recapitulates principles of osteogenesis in an appositional bone growth manner surrounding the implant's surfaces by requiring the activity of bone cells. Thus, dental implant surfaces are expected developing a microenvironment able to trigger intracellular pathways to drive cell adhesion, proliferation, and differentiation [3, 4].

Over the last years, a novel surface containing hydroxyapatite in nanoscale (HAnano®, Promimic, Gothenburg, Sweden) was used to coat titanium implants with already known biological responses [5, 6]. This is a 0.02 *μ*m thin coat with bone-mimicking hydroxyapatite layer using nanotechnology with acceptable hydrophilicity able to have adsorption of blood components and so it favors cell performance, accelerating osseointegration by enhancing appositional bone growth [7, 8]. Technically, the HAnano® surface creates a super hydrophilic surface without changing the microstructure of the dental implants, and this characteristic is also achieved by the sandblasted and acid-etched SLActive® surface (Straumann, Basel, Switzerland) based on its high surface energy, which is described to result in stronger bone responses [9].

In the bioanalysis field, we are pioneer in proposing molecular approaches to access the molecular and cellular machinery of cells responding to different biomaterials and a series of in vitro approaches have been proposed to cover cellular mechanisms such as western blotting, qPCR, colorimetric assays, and analysis of global repertoire of kinases [2, 10, 11]. In turn, kinases are enzymes with transferase activities and able to contextualize cells through intracellular biochemical cascade pathways [12–14]. Considering the biomaterial field, we have explored these methodologies to suggest biomarkers able to predict the quality of the interaction between cell and biomaterial surface, such as cascades of signaling pathways upon integrin activation with pivotal and biphasic role of Src, which coordinates proliferative/survival and differentiation pathways in osteoblasts [15–19]. Previously, we have shown a comparison of intracellular signaling pathways triggered upon integrin activation by different titanium-modified surfaces (nanometer scale-related roughness, dual acid-etching surfaces) and the pivotal role of surface properties on cell behavior is clear. In turn, nanometer scale titanium surface texturing involves Fak and Src as major players during osteoblast viability and differentiation [18].

In order to better address the biological response to HAnano®, osteoblasts were grown on the implant's surface up to 72 hours and compared with osteoblasts responding to the well-known SLActive® titanium surface, considered here as a gold standard and reference control, once SLActive® surface has a high degree of hydrophilicity and optimal bone cell response [20–22]. Summarizing, compared to SLActive®, the biological data obtained in response to HAnano® are very promising in implantology once it reprograms a set of genes involved with osteoblast adhesion, proliferation, and differentiation, as well as stimulating bone turnover-related genes.

## 2. Material and Methods

### 2.1. Implants and Reagents

The titanium-based dental implants evaluated were Straumann BLX with SLActive® surface from Straumann, Basel, Switzerland, and EPIKUT® with HAnano® surface, from S.I.N. Implant System, Sao Paulo, Brazil, both with 3.5 mm diameter and 10.0 mm length. The HAnano® surface relates to synthetic crystalline calcium phosphate, in particular hydroxyapatite producing a coating of nanosized crystalline hydroxyapatite, as detailed previously [7]. Minimum Essential Medium Eagle-*α* Modification (*α*MEM), Fetal Bovine Serum (FBS), trypsin, penicillin, and streptomycin (antibiotics) were purchased from Nutricell (Campinas, Sao Paulo, Brazil). Trypan blue (T6146), acetic acid glacial (695092), 3-(4,5-dimethylthiazol-2-yl)-2,5-diphenyltetrazolium bromide (MTT) (M2128), ethanol (459844), and crystal violet (C0775) were purchased from Sigma Chemical Co. (St. Louis, MO, USA). TRIzol™ Reagent (15596026), DNase I (18068015), and High-Capacity cDNA Reverse Transcription Kit (4368814) were obtained from Thermo Fisher Scientific Inc. (Waltham, Massachusetts, EUA). Gotaq qPCR master mix (A6002) was purchased from PROMEGA (Madison, Wisconsin, EUA). Oligonucleotides for gene expression, microRNA, and promoter methylation were purchased from Exxtend (Campinas, São Paulo, Brazil).

### 2.2. Preosteoblast Cultures

Preosteoblasts, MC3T3-E1 (subclone 4) (ATCC CRL-2593), were cultured in *α*MEM supplemented with 10% of FBS, containing 1% antibiotics (100 U mL^−1^ penicillin,100 mg mL^−1^ streptomycin), ribonucleosides, and deoxyribonucleosides, at 37°C in a humidified atmosphere containing 5% CO_2_ into a conventional incubator. Both cell viability and density were assayed by the trypan blue dye exclusion test, and subcultures were every 3 days.

### 2.3. Conditioned Medium Preparation

The conditioned medium was prepared according to ISO10993-5:2016 and as proposed by us previously to evaluate biomaterials [16, 17, 23]. Briefly, commercially evaluable dental implants were transferred to sterilized conic tubes (at sterile chamber) and incubated into cell culture media (*α*MEM) at a ratio of 0.2 g/mL (*w*/*v*) up to 24 h at 37°C in a humidified atmosphere containing 5% CO_2_ 37°C. Further, the conditioned medium was used to determine the cytotoxicity of those implants.

### 2.4. Cytotoxicity Assay

MC3T3-E1 (subclone 4) (1 × 10^4^ cells per well) were seeded in sextuplicate into 96-well plates, and in semiconfluency, the cultures were challenged with conditioned medium, and after 3, 24, and 72 h of incubation, at 37°C in a humidified atmosphere containing 5% CO_2_, the medium was replaced with *α*MEM without FBS but containing vital dye MTT [24]. After 3 hours of incubation in the incubator at 37°C, the medium containing MTT was aspirated completely and the viable cells were estimated by solubilizing the formazan blue with DMSO, and the absorbance was measured at 570 nm. The analysis was made by expressing the data in percentage of the control cultures (untreated cells).

### 2.5. Cell Adhesion Assay

For evaluating cell adhesion performance, preosteoblasts were trypsinized, properly counted, and then reseeded (1 × 10^4^ cells per well) in sextuplicate into 96-well plates in an implant-conditioned medium supplemented with 10% of FBS and 1% antibiotics up to 24 h. Then, the nonadherent cells were removed by washing with PBS (37°C) and the adherent cells fixed in glacial acetic acid and absolute ethanol solution (3 : 1; *v*/*v*) for 10 minutes at room temperature (RT). Thereafter, the cells were stained with 0.1% (*w*/*v*) crystal violet for 10 minutes at RT. The excess dye was retained by decanting and washing (2x) with distilled water. For reading, the dye was extracted with 10% acetic acid (*v*/*v*) and the optical density measured at 550 nm using a microplate reader (BioTek Co., Winooski, VT). For the positive control, the cells were seeded on a polystyrene surface (control group, Ctrl). The results were expressed as percent of the control (100%).

### 2.6. Scanning Electron Microscopy (SEM)

For SEM analysis, MC3T3-E1 (subclone 4) (5 × 10^4^ cells per well) were plated in a 6-well plate containing the implants and incubated for 24 h. After this time, the cells were fixed with 2.5% of glutaraldehyde in 0.1 M phosphate buffer pH 7.3 for 24 h at 4°C. The samples were then sent for preparation and analysis at the Electron Microscopy Center (IBB-UNESP, Botucatu, Sao Paulo, Brazil). After immersion in 0.5% osmium tetroxide for 40 min, dehydration by a series of alcohols, drying at a critical point, and finally metallization in gold, the samples were studied using a Quanta 200—FEI Company scanning electron microscope at an accelerating voltage of 12.5 kV.

### 2.7. Preparing of qPCR Samples and Gene Expression Analysis

MC3T3-E1 (subclone 4) (5 × 10^4^ cells per well) were plated on implants placed into 6-well plates and incubated up to 72 h (24, 48, and 72 hours). Thereafter, implants containing cells were washed with PBS and the cells collected in Ambion TRIzol Reagent (Life Sciences–Fisher Scientific, Waltham, MA) to collect adherent cells. The total RNA was extracted by using protocol TRIzol/chloroform, and after DNase I treatment (Invitrogen, Carlsbad, CA), cDNA synthesis was performed with High-Capacity cDNA Reverse Transcription Kit (Applied Biosystems, Foster City, CA) according to the manufacturer's instructions. For quantitative polymerase chain reaction (qPCR), the StepOnePlus machine (Applied Biosystems) was used with 40 cycles in reactions in 10 *μ*L containing PowerUp™ SYBR™ Green Master Mix 2× (5 *μ*L; Applied Biosystems), 0.4 *μ*M of specific primers (Table 1), 50 ng of cDNA, and nuclease-free H_2_O. Gene expression was expressed as compared to control cells by the *^ΔΔ^*CT method, using *Actb* and *Gapdh* represented on the plate as housekeeping controls in three independent experiments in triplicate.

### 2.8. Statistical Analysis

All experiments were performed at least three times. Results were expressed as mean ± standard deviation. Statistical analysis was performed by analysis of variance (ANOVA) followed by the post hoc Tukey test when more than two groups were compared, using GraphPad Prism 5 (GraphPad Software Inc., San Diego, CA, EUA). Differences were considered significant at *P* < 0.05 in two-sided tests of statistical significance.

## 3. Results

**Figure 1:**
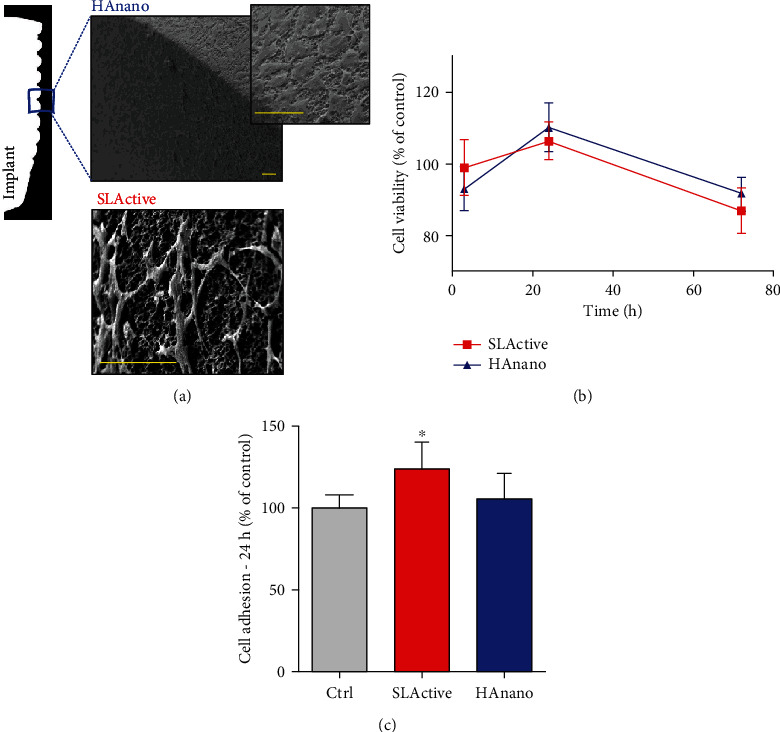
Morphological changes and cell adhesion and viability. Electron micrographs of preosteoblast adhered to the surface of dental implants ((a) bars = 60 *μ*m). Semiconfluent cultures of preosteoblasts were challenged with implant-conditioned medium, and cellular viability was assessed by MTT reduction after 3, 24, and 72 hours (b). Cell adhesion was assessed after trypsinization followed by reseeding of cells with conditioned medium by dental implants and assessed by violet crystal staining after 24 h (c). The cytotoxicity and adhesion data were expressed as percentage of the control group (100%) and represented as mean ± SD of three independent experiments run in sextuplicate. ∗*P* < 0.05 when compared to Ctrl.

We have experimented the behavior of osteoblasts growing on 2 different dental implants (with SLActive® and HAnano® surfaces), as well as compared both sets of data with the cultures grown on polystyrene. Firstly, osteoblasts cultured on HAnano® displayed a well-spread performance on the surface increasing the contact zone with the material (Figure 1(a)), while osteoblasts grown on the SLActive® surface seem to have more fusiform morphology. The cell viability was measured by performing MTT approach, technology enabled to access the mitochondrial performance, and our data shows that there is no cytotoxicity up to 72 hours (Figure 1(b)). Even presenting distinct morphologies, this appearance does not affect the adhesion profile of cells (Figure 1(c)).

**Figure 2:**
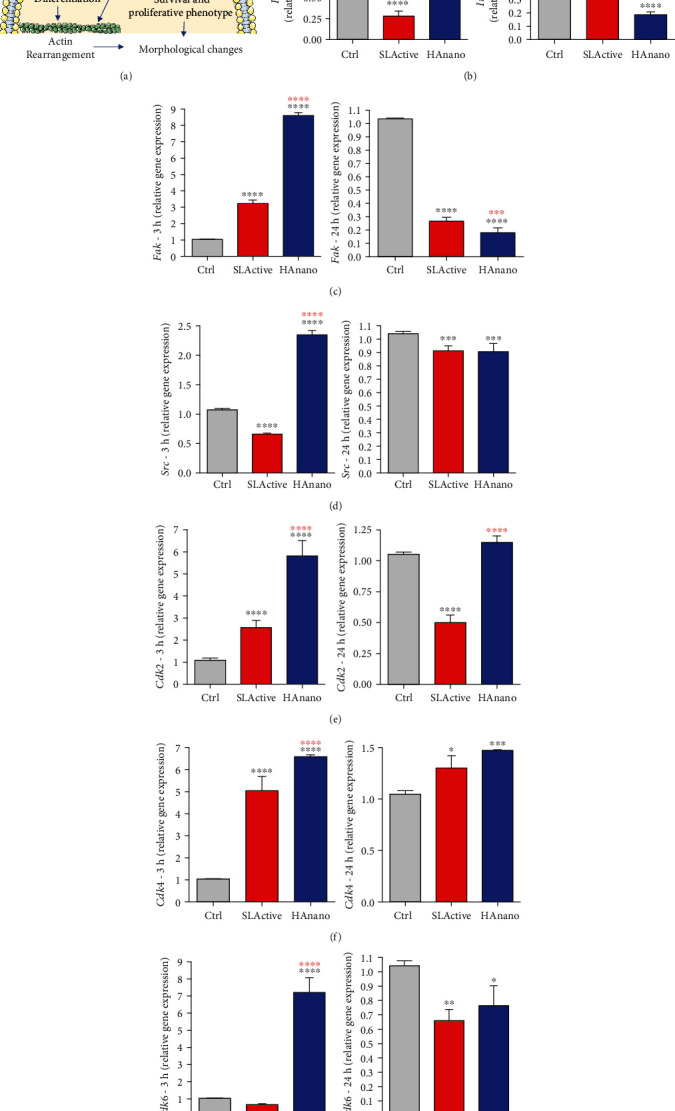
HAnano® triggers intracellular signaling through integrin activation in 3 and 24 hours of attachment. (a) Scheme of the signaling pathway downstream upon integrin activation. Transcriptional profile determination of *integrin* (b) *β1*, (c) *Fak*, (d) *Src*, (e) *Cdk2*, (f) *Cdk4*, and (g) *Cdk6* genes after 3 h and 24 h of osteoblast adhesion by qPCR technology. The relative gene expression levels were determined using the cycle threshold (Ct) method and showed in a graphical format with normalized values as a function of the control assigned value 1. The results represented as mean ± standard deviation of three independent experiments. 3 h: ∗∗∗∗*P* < 0.00001 when compared to Ctrl and ∗∗∗∗*P* < 0.00001 when compared to SLActive®; 24 h: ∗*P* < 0.05, ∗∗*P* < 0.001, ∗∗∗*P* < 0.0001, and ∗∗∗∗*P* < 0.00001 when compared to Ctrl and ∗∗∗*P* < 0.0001 and ∗∗∗∗*P* < 0.00001 when compared to SLActive®.

As it was mentioned before, significant morphological changes were observed in osteoblast adhering on HAnano® and SLActive® and it could be governed by different pathways related with cell adhesion in order to maintain cell viability. Thus, we proposed cell adhesion-related signaling upon integrin activation as possible role in this guidance of osteoblast morphology and viability (Figure 2(a)). These pathways were evaluated up to 3 and 24 hours of osteoblast attachment as follows: at 3 hours, our data shows that there is an upmodulation of the profile of all *integrin β1* (Figure 2(b)), *Fak* (Figure 2(c)), and *Src* (Figure 2(d)) genes by the HAnano® surface, while SLActive® promoted upexpression of *Fak* (Figure 2(c)). Importantly, this cascade of signaling seems to culminate on the activation of genes related to control cell cycle progression, once *Cdk2* (Figure 2(e)), *Cdk4* (Figure 2(f)), and *Cdk6* (Figure 2(g)) were significantly upregulated in response to HAnano®, while SLActive® promotes the activation of *Cdk2* (Figure 2(e)) and Cdk4 (Figure 2(f)); at 24 hours, the profiles of expression of *integrin β1* (Figure 2(b)), *Fak* (Figure 2(c)), and *Src* (Figure 2(d)) genes were significantly downregulated, while the proliferative stimulus remained higher in HAnano® (Figures 2(e)–2(g)).

**Figure 3:**
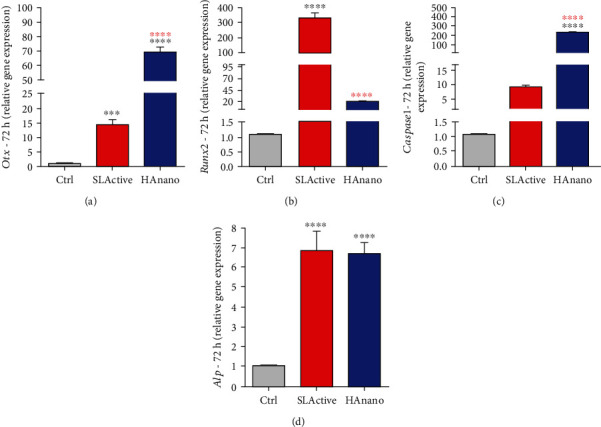
HAnano® stimulates osteogenic phenotype. Transcriptional profile determination of (a) *Otx*, (b) *Runx2*, (c) *Caspase1*, and (d) *Alp* in osteoblasts subjected to implants up to 72 h by qPCR technology. The relative gene expression levels were determined using the cycle threshold (Ct) method and showed in a graphical format with normalized values as a function of the control assigned value 1. The results represented as mean ± standard deviation of three independent experiments. ∗∗∗*P* < 0.0001 and ∗∗∗∗*P* < 0.00001 when compared to Ctrl and ∗∗∗∗*P* < 0.00001 when compared to SLActive®.

Thereafter, we have also investigated whether HAnano® was able to trigger osteogenic stimulus in osteoblasts cultured on implants up to 72 hours.  Figure 3 shows clearly the osteogenic stimulus of both SLActive® and HAnano® surfaces, which activate different transcript factors: SLActive® activates *Runx2* (Figure 3(a)), while HAnano® activates *Osterix* (*Otx*) (Figure 3(b)); and both surfaces lead to the expression of alkaline phosphatase (*Alp*) gene (Figure 3(d)). Additionally, we have investigated the behavior of Caspase1 gene, which has been related with inflammasome and osteoblast differentiation [25, 26]; our data shows that although SLActive® also promotes its expression, it was more significant in response to HAnano® (Figure 3(c)).

**Figure 4:**
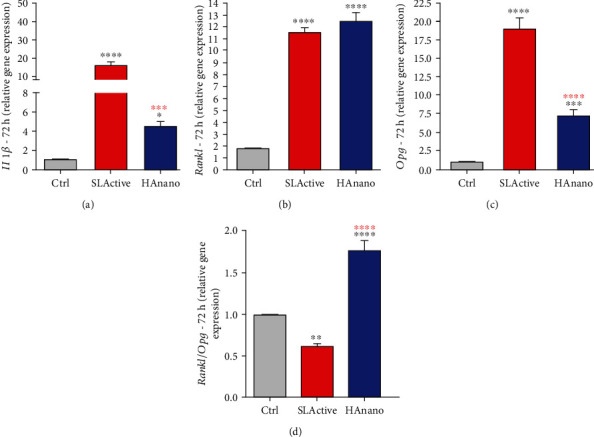
Immunological-related members indicate a stimulus of bone remodeling in response to HAnano® surfaces. Transcriptional profile determination of (a) *Il1β*, (b) *Rankl*, (c) *Opg*, and (d) *Rankl/Opg* ratio in osteoblasts subjected to implants up to 72 h was evaluated by qPCR technology. The relative gene expression levels were determined using the cycle threshold (Ct) method and showed in a graphical format with normalized values as a function of the control assigned value 1. The results represented as mean ± standard deviation of three independent experiments. ∗*P* < 0.05, ∗∗*P* < 0.001, ∗∗∗*P* < 0.0001, and ∗∗∗∗*P* < 0.00001 when compared to Ctrl and ∗∗∗*P* < 0.0001 and ∗∗∗∗*P* < 0.00001 when compared to SLActive®.

Finally, bone-related immunological gene markers were evaluated in osteoblasts responding to both surfaces (Figure 4). Our data shows that both implant surfaces promote *Il1β* upexpression (Figure 4(a)), although it is higher in response to SLActive® (>10-fold changes). Although both SLActive® and HAnano surfaces promote *Rankl* gene expression (both around 12-fold changes) (Figure 4(b)), osteoprotegerin (*Opg*) gene expression was higher in SLActive® (around 17.5-fold changes), while HAnano® promotes the increase of 7.5-fold changes (Figure 4(c)). This difference in *Opg* expression reflects on the *Rankl/Opg* ratio (Figure 4(d)).

**Figure 5:**
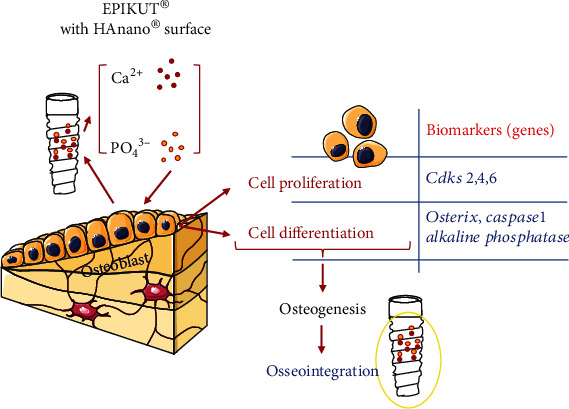
Overview of molecular mechanisms triggered by the HAnano® surface in osteoblasts. This scheme depicts the main biological mechanisms triggered by the HAnano-modified surface in osteoblast responses. When in contact with the surface, osteoblast upmodulates the activity of a set of genes related with cell adhesion at early 3 and 24 hours and further compromises the expression of genes related with osteoblast differentiation. Importantly, calcium and phosphate ions are hypothesized to be released as described within current literature which triggers signaling pathway upstream activating osteoblast proliferation and differentiation. Altogether, these biological stages of osteoblast biology culminate on osteogenesis process and are expected being recapitulated during osseointegration of dental implants.

Figure 5 brings an overview of the main findings obtained in this study, where HAnano® provides an adequate microenvironment/surface to reprogram a set of genes in osteoblasts with the ability to drive their adhesion, proliferation, and differentiation, culminating on the osteogenesis process. These stages of osteogenesis are recapitulated in an appositional bone growth manner during osseointegration of dental implants.

## 4. Discussion

Although titanium presents decisive biological and physicochemical properties, there is an increased effort to propose a novel active surface able to accelerate the osteointegration of dental implants as well as recovering faster physiological and social issues of patients [27]. Among other chemicals, calcium phosphate-based materials have been intensively investigated due to their chemical similarity to bone minerals and potential bioactivity. Over the last years, an alternative of thinner coatings, based on nanoparticles of hydroxyapatite, has proposed adequate microenvironment to support the adsorption of circulating protein as well as cell adhesion and differentiation [6].

In this study, we have better addressed the biological behavior of osteoblasts adhering on a nanoscale hydroxyapatite surface (HAnano®) by comparing these outcomes with those data obtained by a sandblasted and acid-etched titanium surface (SLActive®) considered as a super hydrophilic surface and resulting in good appositional bone growth [20]. Firstly, our data shows there is a differential behavior of osteoblasts adhering on both surfaces; osteoblast spreads better over HAnano®, while osteoblast on SLActive presented a fusiform morphology. It is important to mention that this ability of an adherent cell to spread has important consequences during osteointegration of dental implants, occupying faster the surface of implants and immediately triggering signal for osteoblast differentiation, maybe because HA seems to mimic the nature of inorganic fraction of bone favoring a better performance on promoting cell interaction. Although initial stages guarantee cell contact with the surfaces, later stages involve active processes of actin rearrangement—we have proposed to investigate a program of genes encoding proteins related with downstream signaling upon integrin activation by assembling stable molecular platforms intracellularly and requiring the activity of Fak, Src, and Paxillin in order to know about the quality of substrate/cell interaction.

In this way, this set of genes was evaluated and our data shows clearly that HAnano® promotes a microenvironment able to upmodulate *integrin*, *Fak*, and *Src* genes, supporting an early commitment to drive cell adhesion. Taken these data into account, we can speculate that osteoblasts create platforms of focal adhesion points up to 3 hours of attachment, and this cascade leads the signal to cytoskeleton rearrangement as it was reported previously using other biomaterials [11, 28–30]. The activation of this signaling cascade drives proliferative behavior of osteoblasts, where Src develops pivotal and biphasic roles, once Src is required by both proliferative and differentiation pathways [2, 5, 18, 31–33]. Additionally, there is a decrease on the profile of expression of those adhesion-related genes at 24 hours maybe because these genes already expressed enough transcripts to guarantee the performance of osteoblast adhesion. As this signaling pathway orchestrates cell survival and proliferation, it is expected to be upstream signals to drive the upexpression of genes related with cell proliferation such as *Cdk2*, Ckd4, and Cdk6 in osteoblast responding to the HAnano coating. This signaling pathway seems to be triggered extracellularly by a previously formed coating of serum proteins once calcium forming hydroxyapatite is known to affect the adsorption of extracellular matrix proteins on the surface [34, 35], and this property is decisive to provide ideal extracellular interactions with the integrin domains, upstream member of cell viability, and proliferative-related pathways with biphasic role of Src involvement.

The already known biphasic role of Src in osteoblasts proposes evaluating osteogenic gene markers in a circuit of osteoblast differentiation. Here, our data shows there is differential ability of both surfaces (HAnano® and SLActive®) on promoting the expression of genes related with osteogenic phenotype, but with differential expression profile of *Osterix* and *Runx2* genes: while HAnano® surface requires significant activation of Osterix, SLActive® promotes significantly the upexpression of *Runx2*. Independently, both *Osterix* and *Runx2* signaling culminates on the expression of alkaline phosphatase (*Alp*) gene and osteogenic differentiation. This differential mechanism supporting osteoblast differentiation promoted by both surfaces evaluated in this study can be explained by the probable release of calcium and phosphate amounts to cell culture medium, which regulates the activation of osteoblasts in considering adhesion, proliferation, and differentiation by affecting the expression of classical osteoblastic differentiation markers [36–38] by modulating ERK and PI3K/AKT pathways [39–41]. Importantly, MAPKs are also regulated by phosphate and culminate on cell proliferation and differentiation by increasing the expression of bone morphogenetic proteins (BMPs) [42, 43]. Thus, the presence of HAnano on the surface of dental implants promotes an ideal microenvironment to osteoblast adhesion by dynamically interacting with extracellular matrix proteins providing a coating of protein of their surfaces able to activate integrin located within the cell membrane. With the release of calcium and phosphate to the microenvironment of the implant, it is expected to promote active consequences on osteoblast differentiation and osteogenesis. Importantly, we have shown a solubility constant of hydroxyapatite in aqueous solution and this modification on HA ameliorates the interaction with osteoblasts [11, 44–46]. In addition, it is also known that titanium surfaces are able to adsorb protein from the blood, improving significantly their biocompatibility [47]. Evidently, this hierarchical set of stages of cell involvement during osteogenesis must be recapitulated during the appositional bone growth in osseointegration mechanism.

Lastly, we have also shown that Caspase1 gene was also significantly upmodulated in response to HAnano® and it suggests an involvement of inflammasome during osteoblast differentiation mechanism, as it was suggested previously [25, 26], as well as stimulus for osteoclastogenesis since the Rankl/Opg ratio is increased in osteoblasts responding to HAnano®. However, the effectivity of HAnano® on osteoclast biology needs to be better investigated.

Collectively, this study provides enough evidences to support that the nanohydroxyapatite-coated surface provides necessary microenvironment to drive osteoblast performance on dental implants and these stages of osteogenesis are expected during the early stages of osseointegration.

## Figures and Tables

**Table 1:** Data sheet of the specific genes evaluated in this study.

Gene (ID)	Primer	5′-3′ sequence	Reaction's condition	Product size (bp)
Integrin b1 (16412)	Forward	CTG ATT GGC TGG AGG AAT GT	95°C, 15 s; 63°C, 30 s; 72°C, 30 s	173
	Reverse	TGA GCA ATT GAA GGA TAA TCA TAG		
Fak (14083)	Forward	TCC ACC AAA GAA ACC ACC TC	95°C, 8 s; 61°C, 8 s; 72°C, 8 s	101
	Reverse	ACG GCT TGA CAC CCT CAT T		
Src (17977)	Forward	TCG TGA GGG AGA GTG AGA C	95°C, 8 s; 61°C, 8 s; 72°C, 8 s	134
	Reverse	GCG GGA GGT GAT GTA GAA AC		
Cdk2 (12566)	Forward	TAC CCA GTA CTG CCA TCC GA	95°C, 15 s; 60°C, 30 s; 72°C, 30 s	466
	Reverse	CGG GTC ACC ATT TCA GCA AA		
Cdk4 (12567)	Forward	TCG ATA TGA ACC CGT GGC TG	95°C, 15 s; 60°C, 30 s; 72°C, 30 s	904
	Reverse	TTC TCA CTC TGC GTC GCT TT		
Cdk6 (12571)	Forward	CGC CGA TCA GCA GTA TGA GT	95°C, 8 s; 61°C, 8 s; 72°C, 8 s	325
	Reverse	GCC GGG CTC TGG AAC TTT AT		
Runx2 (12393)	Forward	GGA CGA GGC AAG AGT TTC A	95°C, 15 s; 63°C, 30 s; 72°C, 30 s	249
	Reverse	TGG TGC AGA GTT CAG GGA G		
Osterix (170574)	Forward	CCC TTC CCT CAC TCA TTT CC	95°C, 15 s; 63°C, 30 s; 72°C, 30 s	424
	Reverse	CAA CCG CCT TGG GCT TAT		
Caspase1 (12362)	Forward	TGA AAG AGG TGA AAG AAT T	95°C, 15 s; 63°C, 30 s; 72°C, 30 s	385
	Reverse	TCT CAA GAC ACA TTA TCT		
Alp (11647)	Forward	GAA GTC CGT GGG CAT CGT	95°C, 15 s; 63°C, 30 s; 72°C, 30 s	347
	Reverse	CAG TGC GGT TCC AGA CAT AG		
Gapdh (14433)	Forward	AGG CCG GTG CTG AGT ATG TC	95°C, 8 s; 59°C, 8 s; 72°C, 8 s	332
	Reverse	TGC CTG CTT CAC CAC CTT CT		
*β*-Actin (11461)	Forward	TCT TGG GTA TGG AAT CCT GTG	95°C, 8 s; 60°C, 8 s; 72°C, 8 s	82
	Reverse	AGG TCT TTA CGG ATG TCA ACG		

## Data Availability

The data that support the findings of this study are available from the corresponding author upon reasonable request.
